# Editorial: On the Diversity of Roles of Organic Acids

**DOI:** 10.3389/fpls.2016.01592

**Published:** 2016-10-25

**Authors:** Maria F. Drincovich, Lars M. Voll, Veronica G. Maurino

**Affiliations:** ^1^Centro de Estudios Fotosintéticos y Bioquímicos, Universidad Nacional de RosarioRosario, Argentina; ^2^Division of Biochemistry, Department of Biology, University of Erlangen-NurembergErlangen, Germany; ^3^Institute of Developmental and Molecular Biology of Plants, Plant Molecular Physiology and Biotechnology Group and Cluster of Excellence on Plant Sciences, Heinrich Heine UniversityDuesseldorf, Germany

**Keywords:** light enhanced dark respiration, tricarboxylic acid cycle, organic acids, photorespiration, photosynthesis

Organic acids represent intermediates of major carbon metabolism in plant cells and are, among others, involved in various biochemical pathways, such as glycolysis, the tricarboxylic acid cycle, photorespiration, the glyoxylate cycle, or the photosynthetic C_4_ cycle. During the last years, compelling evidence has pointed out that organic acids also display unexpected roles in controlling whole plant cell physiology. These novel roles have become evident by the contribution of a broad spectrum of research laboratories, which have analyzed plants with modified levels of these compounds and found surprising effects (Zell et al., 2010; Centeno et al., 2011; Penfield et al., 2012; Medeiros et al., 2016).

Organic acids are involved in the regulation of a broad range of basic cellular processes, e.g. the modification of cellular pH or the redox state. Therefore, it makes sense that these compounds also play a role in the control of various biochemical and physiological processes *in vivo*. As extensively analyzed in the case of sugars, a role of organic acids as signaling messengers is emerging (Finkemeier et al., 2013), as well as a role as modulators of the transport across biological membranes (Hedrich and Marten, 1993; De Angeli et al., 2013). Similarly, organic acid metabolism in the cytosol was shown to be involved in abiotic stress responses like cold acclimation (Dyson et al., 2016). Recent evidence even indicates that organic acids are involved in chemical modification of proteins, as for example acetylation or succinylation, with high impact on the *in vivo* protein activity (Zhang et al., 2011; Weinert et al., 2013). All these novel roles for organic acids have moved them into the spotlight of plant cell biochemistry and whole plant physiology. However, different roles of these compounds still remain to be explored which makes this an intriguing topic in plant sciences.

The present Topic contains two original research articles, two reviews and one perspective article, in all of which novel aspects of the metabolism and contribution of organic acids to whole plant physiology are presented.

Li et al. examined the effect of gamma-aminobutyric acid (GABA) application on early growth, net photosynthetic rate, gas exchange, osmoregulation, and enzymatic activities in three different maize cultivars. They found that application of GABA improves maize seedling growth, in association with improved net photosynthetic rate and antioxidant enzyme activities.

Lehmann et al. analyzed the enhanced CO_2_ release of illuminated leaves transferred into darkness (light enhanced dark respiration, LEDR), using species that exhibit different values of LEDR. For this, the authors fed [^13^C]-malate and [^13^C]-pyruvate labeled at specific positions via the xylem stream to the leaves in order to assess the contribution of these organic acids to LEDR. By this approach, they obtained direct experimental evidence about the respiration of both carboxyl groups of malate during LEDR. The results obtained indicate that the two carboxyl groups of malate undergo isotopic randomization by fumarase in the leaves, introducing an additional level of complexity to the interpretation of respiratory substrates.

Igamberdiev and Eprintsev discuss the rate limiting enzymes involved in the metabolism and accumulation of particular organic acids, such as citrate, malate, aconitate, hydroxycitrate, 2-oxoglutarate, malonate, oxalate, and oxamalonate, emphasizing that the function of these molecules can drastically change depending on the pH of the regarded cellular compartment. Moreover, organic acids are able to extensively modify the cellular, subcellular, or extracellular compartment in which they are found, due to their particular chemical properties.

The flexibility of C_4_ plants in using different types of organic acids and C_4_ decarboxylases is discussed in Ludwig, along with the advantages of this plasticity in changing environments. The complexity of the biochemistry and overall biology of C_4_ plants is revised, pointing out different aspects of C_4_ photosynthesis, which remain to be analyzed. This insight will be valuable for the transfer of C_4_traits into C_3_ crops in order to increase yield or mitigating the effects of climate change.

Finally, Engqvist clearly illustrates the limited availability of experimental data for the functional annotation of enzymes involved in organic acid metabolism, especially in crops and forestry trees. In this sense, the over-reliance on bioinformatic predictions for enzyme annotations may lead to mistakes in enzyme annotations across genomes, and may represent missed opportunities to discover enzymes with unique properties. In this scenario, the development of novel high-throughput platforms for functional characterization of enzymes is a key future challenge.

This collection of papers highlights recent advances on different aspects of organic acid research. Together, they contribute to the identification of new and diverse roles of organic acids in plant biochemistry and physiology, pinpointing important areas for future research, such as the role of organic acids as signaling molecules, the emerging more complex biochemistry of C4 plants regarding the use of mixed types of organic acids, and the development of novel high-throughput platforms for the molecular analysis of enzymes involved in organic acid metabolism to improve their functional annotation.

## Author contributions

All authors listed, have made substantial, direct and intellectual contribution to the work, and approved it for publication.

## Funding

This work was supported by the Deutsche Forschungsgemeinschaft through grant EXC 1028 and the EU 7th Framework Programme Cooperation FP7-289582.

### Conflict of interest statement

The authors declare that the research was conducted in the absence of any commercial or financial relationships that could be construed as a potential conflict of interest.
